# Health-related quality of life among children with transfusion-dependent thalassemia: A cross-sectional study in Malaysia

**DOI:** 10.1186/s12955-020-01381-5

**Published:** 2020-05-14

**Authors:** Asrul Akmal Shafie, Irwinder Kaur Chhabra, Jacqueline Hui Yi Wong, Noor Syahireen Mohammed, Hishamshah Mohd Ibrahim, Hamidah Alias

**Affiliations:** 1 Discipline of Social & Administrative Pharmacy, School of Pharmaceutical Science, Universiti Sains Malaysia, Minden, 11800 Pulau Pinang Malaysia; 2 Institutional Planning and Strategic Centre, Universiti Sains Malaysia, Minden, 11800 Penang Malaysia; 3 Pharmacy Department, Sabah Women and Children’s Hospital, Ministry of Health Malaysia, Karung Berkunci No.187, 88996 Kota Kinabalu, Sabah Malaysia; 4 Pharmacy Department, Hospital Kuala Lumpur, Ministry of Health Malaysia, Jalan Pahang, 50586 Kuala Lumpur, Malaysia; 5 Clinical Research Center, Hospital Sultanah Bahiyah, Ministry of Health Malaysia, KM 6 Jalan Langgar, 05460 Kedah Darul Aman, Alor Setar Malaysia; 6 Tunku Azizah Hospital Kuala Lumpur, Ministry of Health Malaysia, Jalan Raja Muda Abdul Aziz, 50300 Wilayah Persekutuan Kuala Lumpur, Malaysia; 7 Division of Research and Technical Support, Ministry of Health Malaysia, Putrajaya, Malaysia; 8 Paediatric Haematology and Oncology Unit, Department of Paediatrics, UKM Medical Centre, Faculty of Medicine, The National University of Malaysia, Wilayah Persekutuan Kuala Lumpur, Malaysia

**Keywords:** Transfusion-dependent thalassemia, Children, Health-related quality of life, PedsQL™ 4.0

## Abstract

**Background:**

The treatment of children with transfusion-dependent thalassemia (TDT) in Malaysia has progressed since 2005. This study provides an updated health-related quality of life (HRQoL) assessment for children with the disorder and the factors affecting the HRQoL.

**Methods:**

A cross-sectional HRQoL survey of Malaysian children with TDT was conducted using the PedsQL™ 4.0 Generic Core Scales. Patients with non-transfusion dependent thalassemia and other haemoglobinopathies were excluded. Parent-proxy and self-reported HRQoL scores were obtained using a multi-stage convenient sampling. The relationship between HRQoL scores and demographic factors were tested using association, correlation and regression analysis.

**Results:**

A total of 368 patients were recruited. The mean (SD) Total Summary Score (TSS) was 80.12(13.87). Predictors for a lower TSS was an increasing age group and the use of dual chelating agents (R^2^ = 0.057, F (4, 359) = 5.40, p = < 0.001). The mean (SD) Physical Health Summary Score (PHSS) was 82.21 (16.82). Predictors of a higher PHSS score was being male, while predictors of a lower score was an increasing age group and parent-proxy reports(R^2^ = 0.075, F (5,358) = 5.80, p = < 0.001). The mean (SD) Psychosocial Health Summary Score (PCHS) was 79.39 (14.81). Predictors for a lower PCHS was the use of dual chelating agents(R^2^ = 0.041, F (1, 362) = 15.60, p = < 0.001). The school functioning score had the lowest mean (SD) score of 69.52(20.92) in the psychosocial dimension.

**Conclusion:**

The HRQoL of TDT children in Malaysia has improved over the last decade owing to the better access in treatment. However, further effort is needed to improve the school functioning dimension.

## Background

Thalassemia is an inherited blood disorder characterised by the absence or decreased synthesis of one or more of the 4 globin chains in a haemoglobin molecule, causing chronic anaemia. It is classified into transfusion dependent thalassemia (TDT) or non-transfusion dependent (NTDT) [1]. The development of severe anaemia in TDT necessitates regular blood transfusions for survival and optimal growth. Over time, regular blood transfusions result in iron overload as the body lacks mechanism to excrete the excess iron. Iron accumulation is toxic and may lead to complications such as heart failure, cirrhosis, liver cancer, growth retardation & endocrine abnormalities. Iron chelators are then used to excrete the excess iron from the body [2]. There is an estimated 4541 thalassemia patients in Malaysia according to the Malaysian Thalassemia Registry [2] and the numbers are hypothesized to have increased as approximately 4.5% of Malaysians are heterozygous carriers of beta thalassemia and are at risk of having a child affected by it [3].

The diagnosis of thalassemia has shown to affect a patient’s quality of life [4]. Physical changes associated with the disease such as the thalassemia facies and stunted growth may affect a child’s confidence and self-esteem, causing them to feel different or stigmatized. The development of complications may further affect a child’s ability to function normally. A child’s daily routine and attendance to school may also be disrupted with frequent hospital visits for blood transfusions, long infusion hours for iron chelation therapy and fatigue brought about by the anaemia [5].

A Health-Related Quality of Life (HRQoL) assessment measures the impact of a disease and its treatment on an individual’s physical and psychological wellbeing. Understanding the impact of TDT on a patient’s HRQoL can help clinicians to better understand the patients’ needs beyond the clinical markers of the disease. HRQoL findings may also aid health policy decision makers to justify changes or improvement needed to the manner in which health care services are delivered to this group of patients to improve their overall outcomes [6].

HRQoL surveys of children with TDT have been carried out in Malaysia [7–9], Thailand [10–12], India [13–15] and the Middle East [16–20]. Although the principles of treatment are similar, HRQoL outcomes are heavily influenced by experiences, beliefs, expectations, cultural differences and access to health care [21], justifying the need for local studies. In 2005, A. Ismail et al. conducted a local survey using the PedsQL™ instrument to compare the HRQoL of thalassemia children with matched healthy controls [7]. Thalassemia patients were shown to have lower ratings of HRQoL (Total summary score = 67.70) compared to the healthy controls (Total summary score = 79.51 [7]. Another local study conducted in 2009 focused on comparing the HRQoL scores of patients who self-reported and parents who proxy-reported using the PedsQL™. In addition, the caregivers’ HRQoL were described using the EQ-5D. The patients’ self-report revealed a lower mean total summary score of 65.35 compared to the proxy scores of 67.20. The pre-transfusion haemoglobin levels were significantly associated with the child’s HRQoL while the number of thalassaemic children and level of education were significant predictors for the caregivers [8]. In 2009, another cross-sectional survey conducted by Sazlina et.al. using the PedsQL™ showed that the predictors of poor physical HRQoL were the absence of treatment (transfusion and chelation therapy) and the presence of side effects from chelation therapy [9]. The significant predictor of psychosocial HRQoL was the duration of time since diagnosis of the disorder [9]. In all three studies, the average total summary score was lower (ranged between 65 to 70 [7–9], compared to the total summary score in neighbouring Thailand during the same time period, which ranged between 75 to 80 [10–12].

The treatment of thalassemia in Malaysia has remarkably progressed since 2009 [22]. Funds and guidelines were made available for clinical management, prevention and screening programmes [2, 23]. In addition to the subcutaneously administered iron chelator desferrioxamine, oral iron chelators such as deferiprone and deferasirox were supplied free of charge throughout public hospitals to increase accessibility of treatment [22–24]. These substantial support on drug cost from the government are expected to improve the HRQoL of Thalassemia patients in the country. The studies that were conducted in Malaysia previously were centred around urban areas with a limited sample size. Information regarding the effect of the different iron chelation therapy on the HRQoL dimensions were scarce, as the treatment option was initially limited to desferrioxamine. The objective of the current study was to assess the current HRQoL of Malaysian children affected by TDT using the PedsQL™ Generic Core Scales. The relationship of demographic factors associated with the outcome of HRQoL in this population will also be described.

## Methods

### Participants and settings

This was a cross-sectional study with face to face interviews conducted among children and adolescents with transfusion-dependent thalassemia in Malaysia. Participants were selected using multi-stage sampling between May 2018 to September 2018. In the first sampling stage, Malaysia is divided into 5 clusters based on its geographical location; the northern states (Penang and Perak), the central states (Selangor, the Federal Territory of Kuala Lumpur and Negeri Sembilan), the southern state (Johore), the east coast states (Pahang, Kelantan and Terengganu) and the east Malaysian states (Sabah and Sarawak). In the second stage, a non-probability sampling was used to sample patients from each region. The inclusion criteria included patients aged 3–18 years old with a diagnosis of TDT, understands the Bahasa Malaysia or English language sufficiently to complete the survey and consented to participation. Patients who have impaired cognitive function, a diagnosis of non-transfusion dependent thalassemia, other chronic diseases not related to transfusion-dependent thalassemia or other hemoglobinopathies were excluded from this study.

The sample size was determined using the formula for estimating a population prevalence. Unfortunately, there is no official statistics reporting the prevalence of thalassemia in Malaysia as of date. Since the prevalence of the disorder is not known, it was assumed to be 50% to ensure adequate sample size, as recommended by Naing et.al [25]. With an estimated thalassemic population of 4541 [2], 95% level of confidence and a 5% precision rate, the estimated sample size required was 355.

### Data collection

A nationwide training of interviewers was conducted by two of the authors prior to data collection. Interviewers were given a set of forms consisting of a patient information sheet, a parental consent form, an age appropriate assent form, a data collection form for sociodemographic data and medical history, and the PedsQL™ Generic Core Scales (GCS) questionnaire. Before the end of the data collection period, the authors visited study sites to randomly validate the data collection forms with the medical records to ensure accuracy of the collected data.

Based on the inclusion and exclusion criteria, patients were screened and selected when they came in for routine follow up. A written parental consent and the child’s assent were obtained upon agreeing to participate. In this study, parents or caregivers were requested to report the HRQoL of children aged between 3 to 12 years of age. Adolescents aged 13 and above were given the choice to self-report their own HRQoL or if they weren’t able to, a parent proxy-report was done.

This study was registered with the National Medical Research Register (NMRR) of Malaysia (NMRR -17-2614-38,966) and was approved by the Medical Research and Ethics Committee (MREC).

### Health related quality of life assessment instrument

The PedsQL™ 4.0 Generic Core Scale (GCS) instrument is used to measure the HRQoL of children aged between 2 and 18 years old. Survey forms are available appropriate to the respondent’s age and it may be self-reported by the child (ages 5–18 years old) or by a proxy (ages 2–18 years old). The reliability, validity and responsiveness of the instrument has been demonstrated in previous studies [26].

The instrument covers multiple dimensions - physical (8 items), emotional (5 items), social (5 items) and school (5 items), with a total number of 23 items. It questions how much of a problem a particular item from a dimension has been for a child in the last 1 month. Each item has a 5-point Likert scale, corresponding to “never a problem”, “almost never a problem”, “sometimes a problem”, “often a problem” and “almost always a problem”. Items are then reverse scored and transformed into a scale of 0 to 100, with higher scores indicating a better HRQoL [27]. The outcome is computed as a mean of all the items answered into the Total Summary Score (TSS). The Total Summary Score can be further divided into the subscales of Physical Health Summary Score (HSS) and Psychosocial Health Summary Score (PCHS). Within the Psychosocial Health Summary Score (PCHS), there are three dimensions of emotional, social and school functioning score.

### Study tool

A structured questionnaire-survey form (PedsQL™ 4.0 GCS) in English and Bahasa Malaysia (the national language of Malaysia) was used. A validation of the questionnaire in Bahasa Malaysia which was translated from the original English version was made by back translation. The questionnaire had been reviewed for content validity and had been used in a previous HRQOL study among paediatric leukaemia patients in Malaysia [28].

### Statistical analysis

Data analysis was performed using Stata Statistical Software: Release 13 [29]. The HRQOL assessment is reported descriptively based on each dimension, the subscales of Physical and Psychosocial Health Summary Score and the Total Summary Score. The mean scores of this study would then be compared to the mean scores of previous studies conducted in Malaysia. The effect size of the differences between the groups were estimated relative to the pooled standard deviation (Cohen’s *d*). Values of (*d* = 0.2–0.5), (*d* = 0.5–0.8) and (*d* > 0.8) correspond to small, moderate and large differences in HRQoL [30].

The association between demographic factors and the HRQoL dimensions were examined using Mann-Whitney U and Kruskal-Wallis tests to determine if the subgroups of each factor were significantly different from one another. Correlation was done using point-biserial (*r*_*pb*_) and Spearman’s Rank (*r*_*s*_) correlation to measure the direction and magnitude of the association between factors and the HRQoL outcome. Categories of correlation was defined as strong (*r*_*pb*_ or *r*_*s*_ > 0.5), moderate (*r*_*pb*_ or *r*_*s*_ = 0.3–0.5) and weak (*r*_*pb*_ or *r*_*s*_ < 0.3). The effect size using Cohen’s *d* would be estimated and interpreted as described above.

To examine the relationship between the demographic factors and the Physical Health Summary Score, Psychosocial Health Summary Score and the Total Summary Score, a stepwise multiple linear regression was conducted.

Statistical significance was set at a value of less than 0.05 for all the above-mentioned analysis.

## Results

The demographic characteristics of the sampled patients are summarized in Table 1. A total of 368 TDT patients were recruited with 51.63% of the respondents being female. The mean age (SD) was 10.59(4.37) years old and the majority consisted of Malay ethnicity (65.85%). In both the parent-proxy and self-reported groups, the largest portion of respondents had a primary or secondary school education level. Out of the 368 patients, 69.84% were proxy-reported. The mean age (SD) at first transfusion was 2.66 (3.01) years old and the mean (SD) number of years they have been receiving transfusion was 7.68 (4.66) years. Only 27.44% of the patients reported at least one iron overload complication, with 15.49% being endocrine complications such as hypogonadism, diabetes or hypothyroid. A majority of the patients were on oral chelation therapy (72.83%), followed by a combination of subcutaneous injection and oral therapy (15.76%) and subcutaneous injection alone (11.41%). The most commonly prescribed oral chelation therapy was oral Deferasirox, with 63.32% of patients receiving this treatment. Only 6.25% of the total patients reported a history of serious adverse event while using iron chelation therapy.

**Table 1:** Demographic and clinical characteristics of study population

Characteristics	N (%)
**Age (years) (*n*** **= 368)**	
2–4.9 years old	36 (9.78)
5–7.9 years old	62 (16.85)
8–12.9 years old	134 (36.41)
13–18.9 years old	136 (36.96)
**Gender (n = 368)**	
Male	178 (48.37)
Female	190 (51.63)
**Ethnicity (n = 368)**	
Malay	242 (65.85)
Chinese	44 (11.92)
Kadazan-Dusun	55 (14.91)
Others	27 (7.32)
**Region (n = 368)**	
Northern (Penang, Perak)	43 (11.68)
Central (Selangor, Federal Territory of Kuala Lumpur, Negeri Sembilan)	76 (20.65)
Southern (Johore)	45 (12.23)
East Coast (Pahang, Kelantan, Terengganu)	104 (28.26)
East Malaysia (Sabah, Sarawak)	100 (27.17)
**Education Level of Parents who completed Proxy-Report (*n*** **= 257)**	
No Formal Education	12 (4.67)
Primary or Secondary School	183 (71.20)
Tertiary Education	62 (24.12)
**Education Level of Children who completed Self-Report (*n*** **= 111)**	
No Formal Education	3 (2.70)
Primary or Secondary School	97 (87.39)
Tertiary Education	11 (9.91)
**Source of survey (n = 368)**	
Self-reported	111 (30.16)
Parent Proxy-reported	257 (69.84)
**Presence of Iron Overload Complication (n = 368)**	
Absent	267 (72.56)
Present	101 (27.44)
Endocrine Only	57 (15.49)
Liver Only	24 (6.52)
Cardiac Only	3 (0.82)
Multi-system	20 (5.43)
**Iron Chelation Therapy Route of Administration (n = 368)**	
Subcutaneous Injection (SC)	42 (11.41)
Oral (PO)	268 (72.83)
Subcutaneous Injection (SC) + Oral (PO)	58 (15.76)
**Iron Chelation Therapy (n = 368)**	
SC Desferrioxamine	42 (11.41)
PO Deferasirox	233 (63.32)
PO Deferiprone	29 (7.88)
SC Desferrioxamine + PO Deferiprone	41 (11.14)
SC Desferrioxamine + PO Deferasirox	17 (4.62)
PO Deferiprone + PO Deferasirox	6 (1.63)
**Number of iron chelating agents (*n*** **= 368)**	
Monotherapy	304 (82.61)
Dual Therapy	64 (17.39)
**History of Serious Adverse Event with Iron Chelation Therapy(n = 368)**	
No	345 (93.75)
Yes	23 (6.25)
**Characteristics (N = 368)**	**Mean (SD)**
Age (years)	10.59 (4.37)
Age at first transfusion (years)	2.66 (3.01)
Number of years receiving blood transfusion	7.68 (4.66)

*N* number, *SD* standard deviation

Table 2 summarizes the mean scores of the PedsQL™ 4.0 dimensions compared to the previous studies conducted in Malaysia. In the current study, the mean (SD) of the Total Summary Score (TSS) was 80.12 (13.87). The Physical Health Summary Score (PHSS) had a mean (SD) of 82.21 (16.82) while the Psychosocial Health Summary Score (PCHS) had a mean (SD) of 79.39 (14.81). On the PCHS subscales, the school functioning dimension scored the lowest with a mean (SD) of 69.52 (20.92), followed by emotional functioning with a mean (SD) of 78.95 (19.11) and social functioning with a mean (SD) of 88.80 (14.02).

**Table 2:** Overview of studies conducted on the HRQoL of Transfusion-dependent Thalassemia children in Malaysia using the PedsQL™ GCS

Study	A. Ismail et al(7)	Sazlina et al(9)	M. Ismail et al(8)	Current Study
**Data Collection Period**	May 2005 to August 2005	July 2008 to July 2009	August 2009 to January 2010	May 2018 to September 2018
**Study Design**	Cross Sectional	Cross Sectional	Cross Sectional	Cross Sectional
**Sample Size**	78	70	75	368
**Number of Sampling Sites, Region**	1 public hospital from the central Malaysian state	3 public hospitals from the central Malaysian state	1 public hospital and 1 university hospital from the central Malaysian state	12 public hospitals from the northern, central, southern, east coast and east Malaysian states
**Mean (SD) Age**	11.95 (4.3)	11.00 (3.33)	Males: 11.9 (3.3) Females: 12.3 (2.8)	10.59 (4.37)
**PedsQL™ Subscales**				
Total Summary Score	68.91 (12.12)	69.8 (13.1)	65.35 (10.57)	80.12 (13.87)
Physical Health Summary Score	69.15 (16.45)	69.5 (16.6)	69.67 (12.51)	82.21 (16.82)
Psychosocial Health Summary Score	67.58 (12.77)	69.9 (14.9)	63.91 (14.65)	79.39 (14.81)
Emotional Functioning Score	68.14 (17.22)	71.9 (17.5)	59.92 (16.83)	78.95 (19.11)
Social Functioning Score	74.29 (18.77)	78.9 (17.8)	78.01 (13.92)	88.80 (14.02)
School Functioning Score	60.14 (12.77)	58.9 (17.9)	50.59 (15.31)	69.52 (20.92)

Values are presented as Mean (Standard Deviation); n, number

Table 3 summarizes the mean scores of the PedsQL™ 4.0 dimensions based on demographic factors. As age increases, the HRQoL mean scores across dimensions decrease, except for the emotional dimension, which improved as patients grew older. The scores between the genders were similar across the dimensions. HRQoL scores were shown to be lower in the group that received blood transfusion for more than a decade and had iron overload complications. Among the categories of iron overload complications, patients with liver and multisystem complications scored lower compared to patients with only endocrine or cardiac complications. Patients on dual chelating therapy scored lower in all dimensions compared to those on monotherapy. When comparing the routes of administration, the patients on combination of subcutaneous desferrioxamine and oral chelator scored lower compared to those on oral chelator only or subcutaneous desferrioxamine only. The mean scores between groups who experienced a serious adverse event with iron chelating therapy did not differ significantly compared to those who have not had an episode of adverse reaction.

**Table 3:** Correlation between PedsQL™ 4.0 HRQoL scores and demographic factors

Demographic	Mean (SD) Scores					
	Total Summary Score	Physical Health Summary	Psychosocial Health Summary	Emotional Functioning	Social Functioning	School Functioning
**All Patients (n = 368)**	80.12 (13.87)	82.21 (16.82)	79.39 (14.81)	78.95 (19.11)	88.80 (14.02)	69.52 (20.92)
**Age (Years)**						
2–4.9 (n = 36)	86.18 (13.15)	93.19 (7.69)	83.60 (15.86)	77.63 (19.43)	94.44 (9.78)	74.27 (37.43)
5–7.9 (*n* = 62)	80.96 (12.62)	83.57 (15.00)	80.10 (14.02)	77.61 (18.18)	91.61 (11.52)	71.08 (19.38)
8–12.9 (*n* = 134)	80.17 (14.28)	81.48 (17.57)	79.73 (14.78)	79.97 (19.13)	89.10 (13.47)	69.89 (19.86)
13–18.9 (*n* = 136)	78.04 (13.82)	79.32 (17.52)	77.57 (14.81)	78.89 (19.60)	85.64 (15.82)	67.53 (15.82)
***p-Value¥***	0.005*	< 0.001*	*0.056*	*0.706*	0.001*	0.012*
Spearman’s Rank Correlation Coefficient*	−0.157*	−0.197*	−0.126*	*0.036*	−0.206*	−0.149*
**Gender**						
Female (*n* = 190)	79.26 (14.23)	80.13 (18.38)	78.95 (14.99)	79.06 (19.89)	88.24 (14.39)	68.87 (14.39)
Male (*n* = 178)	81.05 (13.46)	84.44 (14.70)	79.85 (14.64)	78.82 (18.31)	89.40 (13.62)	70.24 (20.24)
***p-Value¶***	*0.254*	*0.05*	*0.597*	*0.63*	*0.387*	*0.805*
***Point Biserial Correlation***	*−0.065*	−0.128*	*−0.03*	*0.006*	*−0.041*	*− 0.033*
***Effect Size (Cohen’s d)***	*− 0.129*	*− 0.258*	*−0.06*	*0.012*	*−0.083*	*− 0.064*
**Source of survey**						
Parent-Proxy reported (n = 257)	80.52 (13.77)	82.88 (16.44)	79.69 (14.75)	78.94 (18.69)	89.69 (13.42)	69.47 (21.92)
Self-Reported (n = 111)	79.18 (14.14)	80.60 (17.68)	78.67 (15.01)	78.95 (20.21)	86.68 (15.20)	69.66 (18.42)
***p-Value¶***	*0.405*	*0.263*	*0.528*	*0.738*	0.036*	*0.738*
***Point Biserial Correlation***	*−0.043*	*−0.062*	*−0.031*	*0.0003*	*−0.098*	*0.004*
***Effect Size (Cohen’s d)***	*−0.096*	*−0.136*	*− 0.069*	*< 0.001*	*− 0.215*	*0.009*
**Years of Transfusion**						
Less than 10 years (*n* = 242)	81.25 (13.34)	83.51 (16.32)	8045 (14.22)	79.21 (18.43)	90.66 (12.25)	70.53 (21.56)
More than 10 years (*n* = 126)	77.92 (14.65)	79.67 (17.55)	77.31 (15.77)	78.43 (20.47)	85.16 (16.41)	67.59 (19.59)
***p-Value¶***	0.042*	0.045*	*0.08*	*0.969*	0.001*	*0.105*
***Point Biserial Correlation***	0.114*	0.108*	*0.101*	*0.019*	0.186*	*0.067*
***Effect Size (Cohen’s d)***	*0.24*	*0.228*	*0.213*	*0.041*	*0.398*	*0.14*
**Presence of Iron Overload Complication**						
Absent (*n* = 267)	81.00 (13.61)	83.62 (16.21)	80.09 (14.76)	79.17 (19.12)	89.64 (13.52)	70.48 (21.30)
Present (*n* = 101)	*77.78 (14.34)*	78.44 (17.90)	77.52 (14.88)	78.34 (19.21)	86.57 (15.11)	67.01 (19.76)
***p-Value¶***	***0.044***	***0.011***	*0.094*	*0.743*	*0.068*	*0.091*
***Point Biserial Correlation***	***0.103***	***0.137***	*0.078*	*0.019*	*0.098*	*0.074*
***Effect Size (Cohen’s d)***	*0.233*	*0.31*	*0.174*	*0.043*	*0.22*	*0.166*
Endocrine (*n* = 57)	80.12 (13.02)	80.26 (18.45)	80.00 (13.29)	81.91 (18.11)	87.36 (15.39)	70.31 (17.78)
Liver (n = 24)	74.38 (16.57)	74.61 (17.42)	74.31 (17.22)	82.92 (19.67)	86.45 (16.38)	63.54 (24.02)
Cardiac (n = 3)	83.33 (11.16)	83.33 (10.04)	83.33 (12.01)	83.33 (20.82)	86.67 (10.41)	80.00 (18.03)
Multi-system (*n* = 20)	75.44 (14.49)	79.13 (17.24)	74.18 (15.36)	74.8 (19.86)	84.25 (12.80)	61.84 (18.42)
***p-Value¥***	*0.155*	*0.074*	*0.217*	*0.241*	*0.175*	*0.149*
**Number of Iron Chelating Agents Used**						
Monotherapy (*n* = 304)	81.41 (12.87)	83.27 (15.71)	80.76 (13.75)	80.18 (18.14)	90.28 (12.67)	70.95 (20.47)
Dual Therapy (*n* = 64)	73.88 (16.75)	77.03 (20.84)	72.76 (17.84)	72.95 (22.52)	81.61 (17.71)	62.63 (17.71)
***p-Value¶***	0.001*	0.049*	< 0.001*	0.032*	< 0.001*	0.008*
***Point Biserial Correlation***	0.204*	0.14*	0.203*	0.142*	0.233*	0.15*
***Effect Size (Cohen’s d)***	*0.554*	*0.374*	*0.551*	*0.381*	*0.635*	*0.401*
**Route of Iron Chelation Administration**						
Subcutaneous (SC) Only (*n* = 42)	80.03 (12.46)	79.89 (16.33)	80.08 (13.27)	80.85 (20.09)	89.14 (12.49)	69.62 (19.91)
Oral (PO) Only (*n* = 268)	81.65 (12.93)	83.69 (15.70)	80.93 (13.89)	80.09 (18.01)	90.45 (12.66)	71.39 (20.55)
Combination of SC + PO (*n* = 58)	72.94 (16.87)	76.84 (20.92)	71.57 (17.66)	72.11 (22.22)	80.71 (18.10)	60.56 (12.45)
***p-Value¥***	0.001*	0.041*	< 0.001*	0.049*	< 0.001*	0.004*
**Iron Chelation Therapy**						
SC Desferrioxamine (n = 42)	80.02 (12.46)	79.87 (16.33)	80.08 (13.27)	80.85 (20.09)	89.14 (12.49)	69.62 (19.91)
PO Deferasirox (*n* = 233)	81.58 (12.89)	83.64 (15.81)	80.85 (13.78)	80.16 (17.80)	90.43 (12.78)	70.98 (20.66)
PO Deferiprone (*n* = 29)	82.03 (13.57)	85.27 (13.44)	80.96 (14.71)	79.28 (18.59)	90.71 (12.30)	72.63 (20.34)
SC Desferrioxamine + PO Deferiprone (*n* = 41)	76.13 (16.10)	81.46 (19.54)	74.25 (17.67)	76.97 (22.49)	81.67 (18.86)	62.36 (19.98)
SC Desferrioxamine + PO Deferasirox (n = 17)	65.62 (16.76)	66.25 (20.64)	65.41 (16.51)	60.94 (17.52)	78.53 (16.56)	56.76 (24.49)
PO Deferiprone + PO Deferasirox (n = 6)	82.60 (13.70)	78.75 (21.92)	83.89 (16.92)	80.83 (25.96)	90.00 (11.40)	80.83 (18.28)
***p-Value¥***	0.003*	0.013*	0.002*	0.011*	0.007*	0.031*
**History of Serious Adverse Event with Iron Chelation Therapy**						
No (*n* = 345)	80.16 (13.85)	82.22 (16.90)	79.44 (14.74)	78.93 (18.96)	88.82 (13.76)	69.65 (20.80)
Yes (n = 23)	79.62 (14.52)	82.02 (15.76)	78.58 (16.37)	79.09 (21.95)	88.57 (18.04)	67.37 (23.41)
***p-Value¶***	*0.913*	*0.815*	*0.912*	*0.807*	*0.558*	*0.692*
***Point Biserial Correlation***	*0.009*	*0.003*	*0.013*	*−0.002*	*0.004*	*0.025*
***Effect Size (Cohen’s d)***	*0.039*	*0.012*	*0.058*	*−0.008*	*0.017*	*0.109*

Values of scores are presented as Mean (Standard Deviation); *n* number *SC*, subcutaneous, *PO* oral *p-value¥, using Kruskal Wallis Test,* p-value¶ *using Mann Whitney U Test*

Spearman’s Rank Correlation and Point Biserial Correlation coefficients which are bold and marked * indicate *p*-values < 0.05

Effect Size (Cohen’s d Interpreted as *d* = 0.2–0.5 (Small), *d* = 0.5–0.8 (Moderate) and *d* > 0.8 (Large)

Mann Whitney U and Kruskal Wallis tests were done to test the significance of differences between the subgroups in each factor. In TSS and Physical HSS, significant differences were seen between patients of different age groups, number of transfusion years, presence of iron overload complications and the choice of iron chelation therapy (type, route and number of agents used). The Psychosocial HSS only showed significant differences between subgroups in the iron chelation type, route of administration and number of agents used.

A point biserial correlation and Spearman’s Rank correlation was performed to determine the magnitude and direction of association between factors and the HRQoL dimensions. All the factors were only weakly correlated with a *r*_*pb*_ or *r*_*s*_ smaller than 0.3. Age was negatively correlated with all the dimensions except for the emotional functioning score. All other factors had a positive correlation with the dimensions.

The magnitude of differences between the mean scores of 2 groups were compared using Cohen’s *d* for the years of transfusion, presence of iron overload complications, number of iron chelating agents and the history of serious adverse events with iron chelating therapy. Most factors only had small effects (*d* = 0.2–0.5) except for the number of chelating agents. The effect sizes between the monotherapy and dual therapy group were moderate on the total summary score, psychosocial health summary score and the social functioning score.

Using a stepwise multiple linear regression, significant predictors for the PedsQL™ subscales were determined as shown in Table 4. Model for the Total Summary Score with the predictors of age range and the use of dual chelating agents produced R^2^ = 0.057, F (4,359) = 5.4, *p* < 0.001. The model for the Physical Health Summary score with age, gender and the source of the survey produced R^2^ = 0.075, F (5,358) = 5.80, p < 0.001 while the model for the Psychosocial Health Summary Score only had dual chelating agents as its significant predictor, producing R^2^ = 0.041, F (1,362) = 15.60, p < 0.001.

**Table 4:** Multiple linear regression analysis predictors of PedsQL™ subscales

PedsQL™ Subscale	β coefficient	95% Confidence interval	
Total Summary Score (TSS)		81.74	90.62
Age 5–7.9 years	−4.362	−9.967	1.242
Age 8–12.9 years	−5.422	−10.436	− 0.409
Age 13–18.9 years	−6.045	−11.206	− 0.884
Dual Therapy	−6.594	−10.5	−2.689
**Constant = 86.18, R**^**2**^ **= 0.057, F (4, 359) = 5.40, p < 0.001**			
**Physical Health Summary Score (PHSS)**		86.85	101.50
Age 5–7.9 years	− 10.344	−17.076	−3.611
Age 8–12.9 years	−12.515	−18.567	− 6.462
Age 13–18.9 years	−15.883	−22.800	−8.967
Male	4.543	1.149	7.938
Parent Proxy-reported	−2.879	−7.805	2.048
**Constant = 94.18, R**^**2**^ **= 0.075, F (5, 358) = 5.80,*p*** **< 0.001**			
**Psychosocial Health Summary Score (PCHS)**		79.111	82.398
Dual Therapy	−7.997	−11.979	−4.015
**Constant = 80.76, R**^**2**^ **= 0.041, F (1, 362) = 15.60, p < 0.001**			

## Discussion

The diagnosis of Transfusion-dependent Thalassemia (TDT) affects a child’s physical and psychological wellbeing. Understanding how the disease and treatment affects the HRQoL dimensions is imperative in improving the delivery of care beyond the clinical markers of the disease. One of the strengths of this study is the large number of patients sampled from various regions across the country. Table 2 provides an overview of the previous studies conducted on the HRQoL of TDT children in Malaysia using the PedsQL™ GCS compared to the current study. The wider geographical selection and larger sample size (*n* = 368 from 12 different centres) in this study minimises any environmental-related factors compared to the previous studies [7–9] which focused only on the urban population and included a smaller sample size(*n* < 100 from 1 up to 3 different hospitals). The findings of this study would be more generalizable to the other TDT paediatric patients in the country.

In this study, we found that the HRQoL scores of TDT patients were higher across all dimensions compared to the scores of similar studies conducted in Malaysia almost a decade ago as seen in Table 2. The previous studies conducted in Malaysia had recruited children with TDT aged 5–18 years old [7–9], while this study included patients as young as 3 years old. Inclusion of this younger cohort could have contributed to the higher mean scores observed, as younger patients is hypothesized to have less morbidity related to the progression of the disease and treatment as the effects of chronic transfusion has not yet manifested.

However, we believe that the free access to iron chelation therapy, especially oral chelators, improved the compliance of patients and led to higher overall scores. The delivery of care has improved over the past decade. The development of clinical practice guidelines and its distribution across the various centres provides evidence-based guidance for the management of patients [2]. This has ensured that even centres who do not have a dedicated haematologist are able to plan the management of patients based on the recommended blood transfusion targets and also initiating iron chelating therapy as soon as the serum ferritin levels increases beyond 1000 μg/L. Iron chelators funded by the government [23] has allowed for better access to treatment [24]. Prior to 2003, less than 20% of TDT patients received sufficient iron chelation therapy and many died from complications of chronic iron overload [22]. The accessibility and sustained use of subcutaneous Desferrioxamine followed by availability of oral Deferiprone in 2005, and subsequently oral Deferasirox in 2012 at no cost to patients throughout public hospitals in Malaysia have significantly decreased the morbidity and mortality related to iron overload complications. In this study approximately 70% of patients were on oral iron chelators. This progress has certainly improved the compliance to treatment, decreased the burden of pain related to the administration of subcutaneous treatment and decreased the burden of treatment related to the use of the subcutaneously administered iron chelator. In addition to that, the availability of MRI T2* scans have allowed clinicians to detect the development of iron overload in vital organs, allowing them to intensify treatment earlier and thus preventing the development of chronic iron overload complications. These factors combined has contributed to the overall improvement of HRQoL in these patients.

The Psychosocial Health Summary Score was consistently found to be lower compared to the Physical Health Summary Score in previous studies conducted in Malaysia [7–9], Thailand [10–12] or the Middle East [16, 31, 32]. We found that the school functioning dimension had the lowest mean score in this study. This dimension, among others, measures the frequency of absenteeism from school either because of illness or for the reason of seeking treatment. Previous studies in various countries reported similar findings [10, 11, 17, 18, 33]. Children with TDT require blood transfusions every 3 or 4 weeks and this could affect their school attendance. It is also possible that suboptimal blood transfusions received did not improve anaemic symptoms which could disrupts a child’s ability to concentrate in class. However, parameters regarding optimal blood transfusion was not collected in this study to verify if there was a correlation. There is scarce information on whether patients tend to score lower in this dimension because of anaemic symptoms leading to poor performance in school or because of absenteeism from school caused by frequent hospital visits. This is an area worth researching as a patient’s schooling performance may affect their future employment prospects and ability to contribute to society. Meanwhile, lower emotional and social scores can be attributed to the physical changes and limitations in activity brought about by the severe anaemia, impacting self-esteem, body image, involvement in physical or social activities and possibly future employment [34]. Psychosocial well-being is a modifiable factor. A patient’s well-being can be further improved with specialized counsellors and social support groups which help patients develop coping mechanisms as recommended in guidelines [1]. Treatment geared towards the prevention of psychological issues can improve a patient’s mental health and increase their ability to comply to treatment, thus improving the physical health and overall HRQoL. Efforts of forming a multidisciplinary team consisting of parents, school officials and health care providers may benefit in improving this dimension outcome.

In this study, 5.7% of the variability of the TSS were negatively predicted by increasing age and the use of dual iron chelating agents. Predictors of a higher PHSS was being male, while increasing age and parent-proxy report were negative predictors. The use of dual iron chelating agents was a predictor for lower scores in the PCHS. In a study conducted in India by Dhirar et al. [33], 9.6% of the variability in TSS scores were predicted by age, age of onset, frequency of transfusion per month, duration of treatment, number of concomitant medicines, number of comorbidities and the total number of visits per year. In a study conducted in Thailand, 8.9% of the variability in the TSS was explained by the age and the severity of the condition. Other studies found clinical markers such as the pre-transfusion haemoglobin levels [11], frequency of transfusion [8, 10, 13] and serum ferritin levels [35, 36] to be significant predictors of HRQoL. The low R^2^ in this study is explained by the absence of these clinical markers.

Age was a consistent predictor across several studies [11, 33, 36]. Apart from the emotional dimension, the negative correlation and coefficient between age and the HRQoL scores implies that HRQoL worsens as patients grow older. As patients grow older, the burden of treatment may increase with higher volume of blood required, onset of complications and the need for higher dosages of iron chelating therapy, resulting in lower HRQoL. This finding contrasts with the findings of Thavorncharoensap et al. [11] and Sazlina et al. [9] which reported that HRQoL in adolescent patients were better than their younger counterpart. The studies rationalized that as patients grew older, their independence and knowledge about the condition helped them cope better with the disease.

Although age is a non-modifiable factor, the choice of iron chelating therapy (type, route or number of agents) and the prevention of iron overload complication are areas where HRQoL can be improved. The use of subcutaneous infusions nightly may impair patients HRQoL as demonstrated by several studies [10, 11, 33]. These studies demonstrated that patients on a single oral chelator had a higher HRQoL compared to those on combined therapy, which was consistent with the findings in this study. If a chelation therapy is not tolerated well by a patient, adherence to treatment would be challenging. Poor adherence leads to development of iron overload complications which will affect a patient’s physical functioning. The use of subcutaneous therapy is expected to affect the patient physically by limiting their range of activities and increasing their experience of pain and discomfort related to the administration of medicine [37].

Socially, patients may feel self-conscious to use medicines around their peers as they grow older. This may result in them withdrawing socially as they feel ‘different’ and may be emotionally affected by the routine of having to comply to their therapy [37]. While the younger children, who may not have insight to understand the disease, may not cooperate during treatment. Besides, some families are not able to cope with the schedule of administering long hours of subcutaneous injection or simply cannot afford the accessories required for the administration. The current practise here is to prescribe oral Deferasirox monotherapy as first line treatment to all newly diagnosed TDT patients who require chelation therapy. With this progress, we expect that the overall HRQoL will improve and the risk of developing iron overload complications would be minimised. Caregivers & patients should be included in the decision-making process with the health care provider regarding the choice of iron chelator to ensure that the patient is able to adhere to the therapy and the quality of life is not compromised by the therapy.

### Study limitations

This study has few limitations. First, it was not compared to a healthy population of children in Malaysia hence making it difficult to truly estimate the impact of the disease on HRQoL. Secondly, most of the survey was completed by a proxy, as some patients were too young to answer themselves. Since proxy-reported surveys may either underestimate or overestimate HRQoL [8, 13, 18], interpreting findings from this study should take that possibility into account. Thirdly, due to time constraint and the variation in documentation of medical records across the centres, it was a challenge to obtain other clinical parameters such as the serum ferritin and the pre-transfusion haemoglobin level, which could have been used to assess the relationship between clinical outcomes and HRQoL.

## Conclusions

This study shows that HRQoL scores of children with TDT in Malaysia has significantly improved compared to previous studies that were conducted between 2005 and 2010 using the PedsQL™. Efforts to maintain the patient’s access to iron chelating therapy and improving the delivery of care is essential to prevent the development of iron overload complications, leading to better health-related quality of life. However, the psychosocial health, especially the school functioning dimension, requires further attention and efforts for improvement.

## Data Availability

The datasets used and/or analysed during the current study are available from the corresponding author on reasonable request.

## Abbreviations

DFO: Desferrioxamine
DFP: Deferiprone
DFX: Deferasirox
GCS: Generic Core Scales
HRQoL: Health-Related Quality of Life
HSS: Health Summary Score
ICT: Iron Chelation Therapy
MREC: Medical Research and Ethics Committee
NMRR: National Medical Research Registry
PCHS: Psychosocial Health Summary Score
PO: Oral Route
QOL: Quality of Life
SC: Subcutaneous Route
SD: Standard Deviation
TDT: Transfusion-dependent Thalassemia
TSS: Total Summary Score
